# Possible increase in insulin resistance and concealed glucose-coupled potassium-lowering mechanisms during acute coronary syndrome documented by covariance structure analysis

**DOI:** 10.1371/journal.pone.0176435

**Published:** 2017-04-21

**Authors:** Satoshi Ito, Tomohisa Nagoshi, Kosuke Minai, Yusuke Kashiwagi, Hiroshi Sekiyama, Akira Yoshii, Haruka Kimura, Yasunori Inoue, Kazuo Ogawa, Toshikazu D. Tanaka, Takayuki Ogawa, Makoto Kawai, Michihiro Yoshimura

**Affiliations:** Division of Cardiology, Department of Internal Medicine, The Jikei University School of Medicine, Tokyo, JAPAN; Rutgers New Jersey Medical School, UNITED STATES

## Abstract

**Objective:**

Although glucose-insulin-potassium (GIK) therapy ought to be beneficial for ischemic heart disease in general, variable outcomes in many clinical trials of GIK in acute coronary syndrome (ACS) had a controversial impact. This study was designed to examine whether “insulin resistance” is involved in ACS and to clarify other potential intrinsic compensatory mechanisms for GIK tolerance through highly statistical procedure.

**Methods and results:**

We compared the degree of insulin resistance during ACS attack and remission phase after treatment in individual patients (n = 104). During ACS, homeostasis model assessment of insulin resistance (HOMA-IR) values were significantly increased (P<0.001), while serum potassium levels were transiently decreased (degree of which was indicated by ΔK) (P<0.001). This finding provides a renewed paradox, as ΔK, a surrogate marker of intrinsic GIK cascade activation, probably reflects the validated glucose metabolism during ischemic attack. Indeed, multiple regression analysis revealed that plasma glucose level during ACS was positively correlated with ΔK (P = 0.026), whereas HOMA-IR had no impact on ΔK. This positive correlation between ΔK and glucose was confirmed by covariance structure analysis with a strong impact (β: 0.398, P = 0.015). Intriguingly, a higher incidence of myocardial infarction relative to unstable angina pectoris, as well as a longer hospitalization period were observed in patients with larger ΔK, indicating that ΔK also reflects disease severity of ACS.

**Conclusions:**

Insulin resistance most likely increases during ACS; however, ΔK was positively correlated with plasma glucose level, which overwhelmed insulin resistance condition. The present study with covariance structure analysis suggests that there are potential endogenous glucose-coupled potassium lowering mechanisms, other than insulin, regulating glucose metabolism during ACS.

## Introduction

Substantial experimental evidence with animal models supports the potential benefit of glucose-insulin-potassium (GIK) administration in patients with ischemic heart disease (IHD) in general by promoting glucose metabolism. The activation of insulin signaling *per se* has a cardioprotective effect mainly via anti-inflammatory, anti-apoptotic, and provasodilatory properties, as well as by accelerating uptake and utilization of glucose in the heart, which becomes an important preferential substrate for ischemic myocardium [1–4]. As such, the sustenance of glycolytic flux is crucial for maintaining cellular viability and ion homeostasis via Na^+^/K^+^-ATPase activation.

However, many clinical trials with variable results have a controversial impact on GIK given to patients with acute coronary syndrome (ACS) [5–11]. A number of factors may offset the cardioprotective effects of GIK, such as elevated glucose levels and volume overload induced by this cocktail infusion [8]. In addition, the presence of insulin resistance during ACS attack is believed to be a critical reason for this paradox, although few studies have directly evaluated the insulin resistance in the acute phase of ischemic attack [12].

We recently reported a transient decrease in serum potassium (K) level during ischemic attack of ACS compared to remission phase after treatment in individual patients [13]. The degree of the transient K decrease is tightly correlated with glucose level during ischemic attack, independently of the diabetic condition. The findings in that study indicated the presence of intrinsic glucose-coupled K lowering mechanisms, like GIK, but without involving insulin, that are activated during ACS attack. Although the study suggested that the degree of transient K decrease, as a parameter of intrinsic GIK cascade activation, represents validated glucose metabolism during ischemic attack, little is known about the degree of insulin resistance at the acute phase of ACS attack.

Several methods have been developed for assessing insulin resistance, including plasma insulin level and homeostasis model assessment of insulin resistance (HOMA-IR). When evaluating the correlation of transient K decrease with glucose and various parameters of insulin resistance, it is logical to simultaneously include every possible factor in the same analysis. However, given that HOMA-IR can be confounded by serum glucose and insulin levels, multiple regression analysis cannot be conducted at the same time. A covariance structure analysis is therefore useful for understanding how relationships among observed variables might be generated by hypothesized latent variables in many areas [14]. The path model, a platform of the covariance structure analysis, is proposed based on the scientific knowledge, abundant experience, steady concept, and clear direction of the study.

To identify the mechanism of GIK tolerance and elucidate other intrinsic compensatory systems for glucose metabolism during ACS attack, we examined whether or not insulin resistance is increased in the acute phase of ischemia and evaluated the relationship between the degree of transient K decrease and insulin resistance as well as other clinical factors related to glucose/electrolytes metabolism using covariance structure analysis.

## Methods

### Study patients

Patients with ACS who required emergency admission to The Jikei University Hospital from September 2014 to August 2016 were included in this study. The ethics committee of The Jikei University School of Medicine approved the study protocol (27–103[7988]). ACS was defined as the presence of myocardial infarction (MI) or unstable angina pectoris, as described in detail previously [13]. Briefly, the presence of any two of the following three criteria was required for the diagnosis of MI: (1) a history of cardiac chest pain lasting at least 30 minutes; (2) typical electrocardiographic changes; (3) an increase in serum creatine kinase (CK) level. Unstable angina pectoris was diagnosed according to the criteria for the Braunwald clinical classification without a serum CK increase. All of the patients were admitted to the hospital and underwent emergent cardiac catheterization within 24 hours of the onset. During the study period, 122 patients were treated for ACS in total. Patients who were receiving or starting dialysis (n = 7), were taking potassium-controlling agents (n = 2), were treated with insulin (n = 8) and/or steroids (n = 6) were excluded. No patient with ACS died from any cause during hospi talization in the present study. Based on these selection criteria, 104 consecutive patients, including 53 with MI (51.0%), were enrolled in the present study (Table 1).

**Table 1:** Clinical characteristics (n = 104).

Age, years	63±13
Male, gender (%)	85(81.7)
Height, cm	166±7.6
Weight, kg	68.4±15.0
BMI, kg/m2	24.7±4.7
BP, mmHg	
Systolic	137±23
Diastolic	78±14
Mean	102±15
K during ischemic attack, mmol/L	3.9±0.4
Na during ischemic attack, mmol/L	139±2.4
eGFR, mL/min/1.73m^2^	76.0±19.0
Cr, mg/dL	0.8±0.2
HbA1c, %	6.1±0.8
Glucose, mg/dL	143±40
Insulin, μU/mL	13.4±10.0
BNP, pg/mL	67±132
LVEF, %	55±9.3
Duration of hospital stay, days	10.7±7.8
Myocardial infarction (%)	53(51.0)
Unstable angina (%)	51(49.0)
Diabetes mellitus (%)	35(33.7)
Hypertension (%)	72(69.2)

BMI: body mass index, BP: blood pressure.

K: potassium, Na: sodium.

Cr: creatinine, eGFR: estimated glomerular filtration rate.

BNP: B-type natriuretic peptide.

LVEF: left ventricular ejection fraction.

### Data collection

The clinical characteristics were collected retrospectively from the hospital medical records. ΔK, indicating the degree of transient K decrease during ischemic attack, was calculated from the difference between K during remission phase (the serum K level at the time of discharge) and K during ischemic attack (the serum K level at the time of the emergent cardiac catheterization), as described in detail previously [13]:

ΔK = K during remission phase − K during ischemic attack

All other biochemical data, including plasma glucose level and insulin level, were measured both at the time of the emergent catheterization and at discharge, except for peak CK level. HOMA-IR (as a parameter of insulin resistance), homeostatic model assessment beta cell function (HOMA-β), and the estimated glomerular filtration rate (eGFR) were calculated as described previously [13, 15, 16]. The definition of Diabetes mellitus (DM), hypertension, and dyslipidemia were described previously [15, 17, 18]. The hemodynamic parameters, including left ventricular ejection fraction (LVEF), were measured at the time of cardiac catheterization [19].

### Definitions of the medication profiles

As for the involvement of renin-angiotensin-aldosterone system inhibitors (RAAS-I) and diuretics, the influence of the changes in each medication profile was examined as follows (also as described previously [13]): “newly-administered (+)” indicates that the medications were not being taken at admission but were introduced during hospitalization; “newly-administered (-)” indicates all the other patients who were not included in newly-administered (+) subject. Two patients in each group were taking the medications at admission but discontinued them during hospitalization, and they were designed as newly-administered (-) subjects.

### Statistical analysis

Continuous variables were expressed as the means ± standard deviation (SD). To compare the serum K, glucose, insulin, BNP levels as well as HOMA-IR and HOMA-β between groups, the statistical analyses were performed using a paired sample t-test. The influence of β-blocker use at admission on ΔK was assessed using one way analysis of variance (ANOVA), and the influence of changes in the medication profiles of RAAS-I and diuretics was evaluated using Mann-Whitney’s U test. To assess the determinants of ΔK, multiple regression analyses were performed after simple regression analyses, as described previously [13]. The patients’ mean blood pressure, plasma glucose level, plasma insulin level, HOMA-IR, HOMA-β during ischemic attack and remission phase, glycohemoglobin (HbA1c), sodium (Na) level, eGFR, B-type natriuretic peptide (BNP), LVEF, and the changes in the medication profile of RAAS–I and diuretics were included as variables. In the multiple regression analysis, indicator variables were employed as follows (also as described previously [13]): one indicator variable coded as 0/1 for variables with two categories (changes in medication profiles of RAAS-I and diuretics) was generated. To evaluate the association of ΔK with the disease severity, all of the patients were divided into two groups based on the median value of ΔK, as described previously [13], and also based on quartiles of ΔK. The ΔK threshold for the incidence of MI was assessed using receiver operating characteristic (ROC) curve, and the optimal cut-off point was defined as a combination of the highest sensitivity and specificity. Continuous variables were evaluated using Mann-Whitney’s U test, and the Chi-square test for categorical variables. All of the data were statistically analyzed using the SPSS Statistics software program (version 22.0, SPSS Inc., Chicago, IL, USA). P < 0.05 was considered to be statistically significant.

### Covariance structure analysis

To confirm the contribution of the plasma glucose levels to ΔK, we performed a covariance structure analysis as in our previous study [14]. This analysis compares the power among the multiple independent variables, which confound each other. A path model based on covariance structure analysis was proposed to investigate the relationship among clinical factors in this study population and specifically to identify probable causal effects on ΔK. The causality model defines some hierarchical regression models between clinical factors and ΔK. Another path analysis was used to investigate the relationship (causality) between RAAS-I newly-administered (+) and peak CK, as a surrogate marker of the disease severity of ACS. The path analysis was performed using the IBM SPSS AMOS software program (version 23, Amos Development Corporation, Meadville, PA, USA). The obtained structural equation models were tested and confirmed at a significance level of P < 0.05.

## Results

### Characteristics of the study patients

Table 1 showed the clinical characteristics of the 104 patients. A total of 33.7% of the patients were diagnosed with type 2 DM, while the mean HbA1c was 6.1±0.8% and the mean HOMA-IR during remission phase was 2.4±3.0 (normal level is < 1.6) (Table 2), indicating high incidence of glucose intolerance in the subjects with ACS. The mean serum K level during ischemic attack was significantly decreased compared to K during remission phase (Table 2). In contrast, both the mean plasma glucose and the mean insulin levels were significantly higher during ischemic attack than during remission phase, leading to a higher HOMA-IR value during ischemic attack (Table 2). However, HOMA-β during ischemic attack was lower than that during remission phase, albeit non-significantly (P = 0.058). These data as well as the time course profiles of the plasma glucose and insulin levels in an individual patient (S1 Fig) indicate that insulin resistance is highly prevalent in patients with ACS and further increased during acute phase of ACS attack. However, concerns remained that the data gathered during ischemic attacks were obtained from patients under various dietary conditions. Therefore, a sub-analysis was performed using the data of the subjects under fasting condition, as confirmed by their medical history. Twenty-three out of 104 patients were determined as in “a definite fasting condition” (>9 hours had passed from the last meal), including subjects with early-morning onset of ACS after overnight fasting (same condition as the blood test for the remission phase). After eliminating the effects of various dietary conditions, we found that the plasma glucose, insulin and HOMA-IR levels were significantly higher and the K level significantly lower during ischemic attack than during the remission phase in these 23 patients (S1 Table). These data are in absolute agreement with the original data from the total 104 patients (Table 2), confirming that insulin resistance is likely to be increased during ischemic attack.

**Table 2:** The comparison of the data during ischemic attack and remission phase (n = 104).

	Ischemic attack	Remission phase	P
K, mmol/L	3.9±0.4	4.3±0.3	<0.001
Glucose, mg/dL	143.0±40.0	109.5±32.2	<0.001
Insulin, μU/mL	13.4±10.0	8.4±6.4	<0.001
HOMA-IR	5.1±5.0	2.4±3.0	<0.001
HOMA-β	65.3±41.9	75.1±55.2	0.058
BNP, pg/mL	67.2±132.0	92.4±129.8	0.116

HOMA-IR: homeostasis model assessment of insulin resistance.

HOMA-β: homeostatic model assessment beta cell function.

### Clinical factors affecting the degree of the transient K decrease (ΔK) during ACS attack

To evaluate the determinants of ΔK, we performed a simple regression analysis (Table 3). Plasma glucose level during ischemic attack, but not that during remission phase, showed a significantly positive correlation with ΔK (P = 0.004). On the other hand, LVEF showed a negative correlation with ΔK (P<0.001). ΔK was not associated with HbA1c, the insulin level, HOMA-IR, or HOMA-β during either ischemic attack or remission phase.

**Table 3:** The results of a simple regression analysis of ΔK (n = 104).

Explanatory variables	Standard regression coefficients	Standard error	F	P
Glucose during ischemic attack	0.280	0.001	8.680	0.004
during remission phase	-0.006	0.001	0.004	0.952
HbA1c	-0.068	0.059	0.478	0.491
Na	-0.030	0.019	0.095	0.759
eGFR	-0.039	0.002	0.153	0.696
BNP	-0.011	0.0003	0.012	0.913
LVEF	-0.367	0.004	15.924	<0.001
Insulin during ischemic attack	0.098	0.004	0.992	0.322
during remission phase	-0.153	0.007	2.450	0.121
Blood pressure (mean)	0.139	0.003	2.003	0.160
HOMA-IR during ischemic attack	0.134	0.009	1.871	0.174
during remission phase	-0.150	0.015	2.334	0.130
HOMA-β during ischemic attack	-0.088	0.001	0.792	0.376
during remission phase	-0.076	0.001	0.600	0.440

ΔK = K during remission phase − K during ischemic attack.

Next, we investigated the influence of the changes in each medication profile on ΔK. Detailed information concerning the medication profiles is shown in S2 Table. Of the 104 patients, 34 (32.7%) were taking one or more RAAS-I and/or diuretics at admission, and 82 (78.8%) had taken these agents at the time of discharge. Eighteen patients (17.3%) were taking β-blockers at admission (β1 selective β-blockers: 10 patients; non-selective β-blocker: 8 patients) and 60 (57.7%, β1 selective β-blockers: 13; non-selective β-blocker: 47) had taken them at the time of discharge. The “newly-administered (+)” group for RAAS-I and diuretics during hospitalization showed a significantly larger ΔK than the “newly-administered (-)” group (S2A and S2B Fig, respectively, P<0.01). The profiles of β-blocker use shown in S2C Fig had no influence on ΔK, at least for the current data, although the number of the patients taking β-blockers during ischemic attack was relatively small.

We next performed a multiple regression analysis in order to assess the independent determinants of ΔK (Table 4). Neither LVEF nor the medication profile influenced ΔK in this analysis. Of note, the plasma glucose level during ischemic attack was the sole independent factor positively correlated with ΔK (P = 0.026), consistent with our previous study [13], whereas HbA1c and insulin levels during ischemic attack had no impact (Table 4). In addition, neither HOMA-IR nor HOMA-β was correlated with ΔK (S3 and S4 Tables, respectively). However, there was a significant positive correlation between plasma glucose level during ischemic attack and HbA1c, insulin, and HOMA-IR (P<0.001, in a linear regression analysis, data not shown).

**Table 4:** The results of a multiple regression analysis of ΔK (n = 104).

Significant variables	Standard regression coefficients	Standard error	P
Glucose during ischemic attack	0.260	0.001	0.026
HbA1c (NGSP)	-0.144	0.061	0.168
Insulin during ischemic attack	-0.030	0.005	0.769
Na	0.078	0.018	0.421
eGFR	0.021	0.002	0.826
LVEF	-0.208	0.005	0.056
RAAS-I newly administered	0.199	0.093	0.060
Diuretics newly administered	0.080	0.167	0.420

Dependent variable: ΔK

Explanatory variables: Glucose, HbA1c, Insulin during ischemic attack, Na, eGFR, LVEF.

RAAS-I or diuretics newly administered.

### Concept of the proposed path model (A)

**Fig 1:**
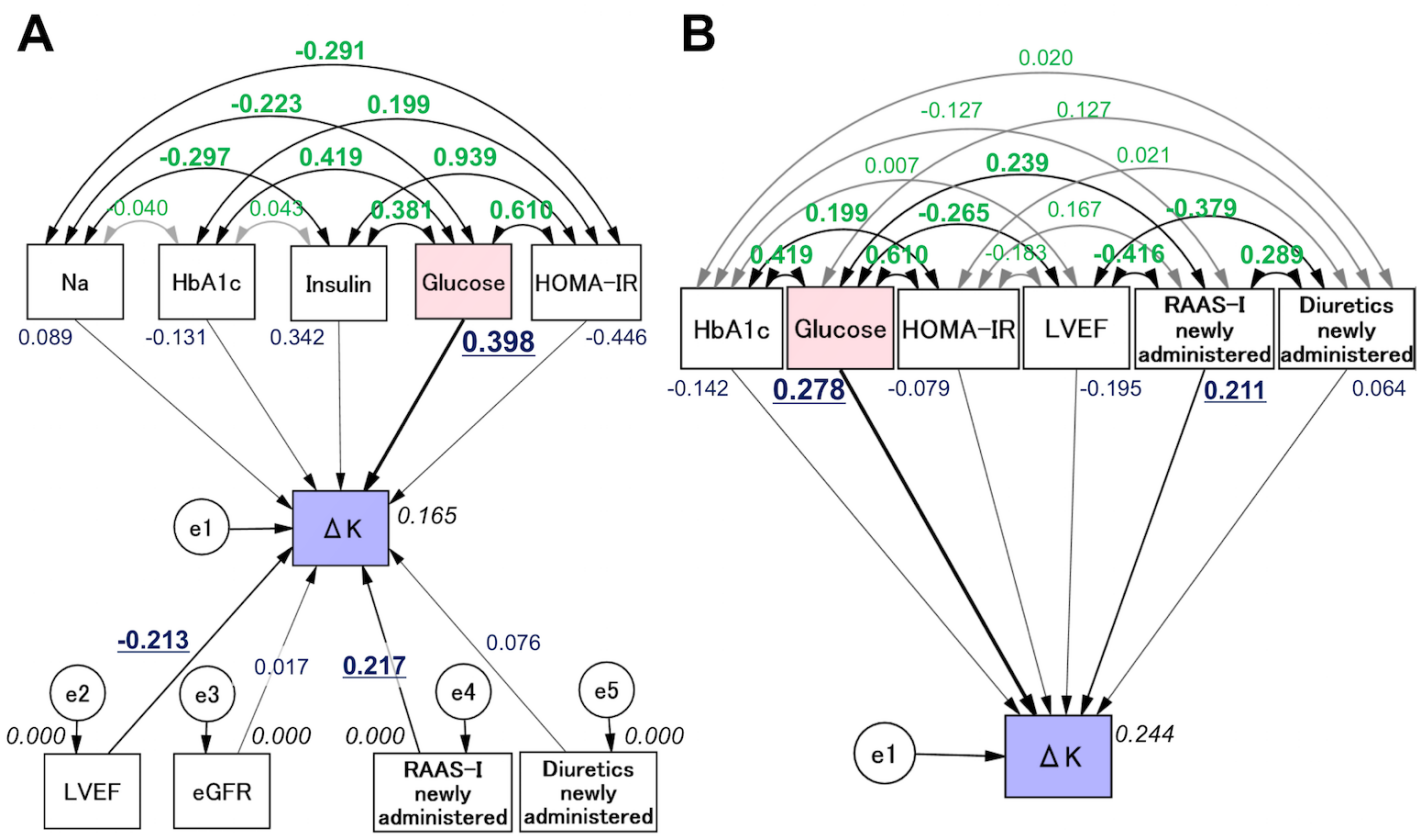
Path models. **A.** Path model theoretically proposed. **B.** Path model modified for the best fit. Each path has a coefficient showing the standardized coefficient of a regressing independent variable on a dependent variable of the relevant path. These variables mean standardized regression coefficients (direct effect) [underlined portions indicate remarkable values], squared multiple correlations [in narrow italics] and correlations among exogenous variables [green].

The proposed theoretical path model is shown in Fig 1A. The path model was created with the blood levels of Na, HbA1c, insulin, glucose, and HOMA-IR in parallel. The correlation between any two factors among the blood levels of Na, HbA1c, insulin, glucose, and HOMA-IR is indicated using two-way arrows. In addition, LVEF, eGFR, and the usage of RAAS-I and diuretics were included as potential factors affecting ΔK. The paths between variables were drawn from independent variables to dependent variable with directional arrows for every regression model—namely, from the blood levels of Na, HbA1c, insulin, glucose, and HOMA-IR to ΔK.

### Results of the path model (A)

The results of the statistical analysis are shown in Table 5. The final model showed the following regression weights after standardizing all variables: ΔK was estimated by the glucose level (standardized regression coefficients, β: 0.398, P = 0.015), LVEF (β: -0.213, P = 0.018) and newly-administered RAAS-I (β: 0.217, P = 0.016). Of note, the plasma glucose levels, but not HbA1c, insulin, or HOMA-IR levels, were associated with ΔK in the covariance structure analysis, which reinforced the findings of the multivariate analysis.

**Table 5:** The results of standardized regression coefficient analysis to identify the clinical factors influencing ΔK in each path model.

		Clinical factor	Direct effect	P
(A) Path model theoretically proposed				
ΔK	**←**	Glucose	0.398	0.015
	**←**	HbA1c (NGSP)	-0.131	0.203
	**←**	Insulin	0.342	0.371
	**←**	HOMA-IR during ischemic attack	-0.446	0.311
	**←**	Na	0.089	0.351
	**←**	LVEF	-0.213	0.018
	**←**	eGFR	0.017	0.849
	**←**	RAAS-I newly administered	0.217	0.016
	**←**	Diuretics newly administered	0.076	0.397
(B) Path model modified for the best fit (removed “Na and insulin and eGFR”)				
ΔK	**←**	Glucose	0.278	0.024
	**←**	HbA1c (NGSP)	-0.142	0.148
	**←**	HOMA-IR during ischemic attack	-0.079	0.467
	**←**	LVEF	-0.195	0.052
	**←**	RAAS-I newly administered	0.211	0.033
	**←**	Diuretics newly administered	0.064	0.494

The results (direct effect) of the path model theoretically proposed (A) and the path model modified for the best fit (B) analysis to identify the clinical factors influencing ΔK (see Fig 1).

The standardized direct effect of each variable on ΔK after standardizing all variables.

### Concept of the proposed path model (B)

In the path model (A), the ΔK was estimated by glucose level; however, we made no allowance for a possible connection between ΔK and the use of drugs or LVEF. To examine whether they were separate effects or not, another theoretical path model (B) was proposed, as shown in Fig 1B. This path model was created with the blood levels of HbA1c, glucose, HOMA-IR, LVEF, and the usage of RAAS-I and diuretics in parallel, which were potential factors affecting ΔK.

### Results of the path model (B)

The results of the statistical analysis are shown in Table 5. The final model showed the following regression weights after standardizing all variables: ΔK was estimated by glucose level (standardized regression coefficients, β: 0.278, P = 0.024) and newly-administered RAAS-I (β: 0.211, P = 0.033). Of note, the effects of glucose and RAAS-I on ΔK can be separately considered as influential factors.

### Clinical significance of the transient K decrease during ACS

To evaluate the clinical implications of ΔK during ACS, we examined the association of ΔK with the disease severity and clinical course. The patients who underwent coronary bypass surgery were excluded from the analysis of “Duration of hospital stay”, since the surgery affects the hospitalization period independently of the disease severity. When all of the study subjects were divided into two groups based on the median value of ΔK (<0.3 and ≥0.3), a higher incidence of MI relative to unstable angina pectoris, in association with a longer hospitalization period and higher peak CK level, was observed in patients with a larger ΔK (Table 6), consistent with the findings from our previous study [13]. We further investigated the association of ΔK with the disease severity and clinical course in the subjects divided into two groups based on quartiles of ΔK (S5 Table). The incidence of myocardial infarction in the subjects with larger ΔK values was consistently higher, at whichever point the quartiles were divided (S5 Table). The ΔK cut-off value for predicting the incidence of MI based on the ROC curve, was 0.3 mmol/L (S3 Fig). These data indicate that ΔK reflects the disease severity of ACS.

**Table 6:** The impact of ΔK on disease severity and clinical course.

	ΔK < median value	ΔK ≥ median value	
Myocardial Infarction	11 (28.2%) [39]	42 (64.6%) [65]	P = 0.001
Peak Creatine Kinase (U/L)	639.8±969.2 [39]	1602.4±2093.0 [65]	P = 0.004
Duration of hospital stay (days)	7.2±4.2 [38]	11.6±7.2 [63]	P<0.001

Median value of ΔK = 0.3.

The number of cases is noted in square brackets.

### Clinical implications of newly-administered RAAS-I on the disease severity of ACS

Given that newly-administered RAAS-I showed a larger ΔK and that ΔK reflects the disease severity of ACS, we can infer that newly-administered RAAS-I *per se* increases the severity of ACS. In order to examine the correlations between newly-administered RAAS-I and the disease severity, another theoretical path model (C) was proposed (S4 Fig).

### Results of path model (C)

The results of the statistical analysis are shown in S6 Table. The final model showed the following regression weights after standardizing all variables: the peak CK was estimated by glucose level (β: 0.437, P = 0.002), indicating that the plasma glucose level is elevated in association with the severity of ACS. This result is consistent with one of the major findings of the present study that glucose level was positively correlated with ΔK, which reflects the disease severity. On the other hand, the peak CK was not estimated by newly-administered RAAS-I (β: -0.349, P = 0.374), whereas newly-administered RAAS-I was estimated by peak CK (β: 0.676, P = 0.010). These data suggest that RAAS-I tends to be administered more frequently in patients with severe myocardial damage, but newly-administered RAAS-I *per se* does not induce myocardial damage. In fact, path model (C) might reflect the clinical settings wherein patients with ACS with a relatively severe condition, such as myocardial infarction with LV dysfunction, need to be administered RAAS-I for the inhibition of myocardial remodeling as well as for other cardioprotective effects.

## Discussion

Impaired glucose tolerance is a common feature in patients with IHD in general and is well established as a risk factor for increased morbidity and mortality [12, 20, 21]. In the subjects of the present study, insulin resistance (as indicated by HOMA-IR) was relatively high in remission phase (namely, stable phase), consistent with the findings from a previous study [12]. We thus clearly showed for the first time that insulin resistance is likely to be increased in the acute phase of ACS attack. Regarding development of insulin resistance, miscellaneous environmental factors, such as a Western-style diet and a sedentary lifestyle, increase insulin resistance, where glucotoxicity and lipotoxicity as well as chronic inflammation are deeply involved [22]. When insulin resistance progresses gradually, these factors play central roles in the coronary endothelial dysfunction, leading to coronary spasm and thrombus formation, both of which can induce ACS attack [23]. It is of clinically importance, an additional physical and mental stresses and high anxiety can become the final straw, inducing an attack. We therefore believe that the rapid increase in insulin resistance at the acute phase observed in the current study could be not only a consequence but also likely a cause of ACS.

We subsequently found that a transient K decrease during ACS attack, the degree of which (as indicated by ΔK) was positively correlated with glucose, was still preserved even under insulin resistance condition. These results indicate that there are potential neurohumoral mechanisms, like GIK but other than insulin, promoting glucose metabolism during acute ischemic attack. The current results were obtained from not only conventional statistical analyses but also a covariance structure analysis, which is a highly statistical procedure. The path model (A) showed a significant influence of glucose on ΔK by taking all of the potential cofounding factors of blood sampling data into consideration. We additionally found that LVEF and a hyperkalemic effect of RAAS-I were also involved in the ΔK regulation in this model. We then developed another path model (B), which again successfully showed a significant influence of glucose on ΔK while making allowances for other confounding factors, including RAAS-I. These data strongly reinforced the results obtained in the multiple regression analyses.

Under high-glucose condition, insulin stimulates an intracellular K shift into the cardiac and skeletal muscles via sodium-proton exchanger (NHE)–Na^+^/K^+^-ATPase activation, leading to a decrease in serum K level [13, 24, 25]. Thus, ΔK assessed in the current study can be considered a surrogate marker of endogenous GIK cascade activation during ACS attack, allowing that insulin resistance is rapidly increased. The possible “insulin-independent” glucose-coupled K-lowering mechanisms are as follows: 1) Elevated catecholamines stimulate Na^+^/K^+^-ATPase primarily via β2-adrenergic receptor [25–27], although the profiles of β-blocker use had no influence on the ΔK values, at least in the current study. However, the dosages of β-blockers might be critical for evaluating their effects on ΔK, and we speculate that an adequate amount of β-blockers might effectively decrease the ΔK during ACS. Since the number of the patients taking high-dose β-blockers was limited in the present study, future analyses concerning the dose dependent effects of β-blockers on ΔK should be performed in a larger population. 2) The activation of renin-angiotensin-aldosterone system in response to ischemic stress might be another mechanism regulating K homeostasis during ACS [28]. 3) We recently reported that sodium-glucose cotransporter (SGLT), which is activated by Na^+^/K^+^-ATPase, significantly contributes to glucose uptake in the heart as the initial rate-limiting step for cardiac metabolism during ischemia-reperfusion injury [4]. There are two major “insulin-independent” glucose transporters in the heart: SGLT1 and GLUT1 [29]. In contrast to facilitated energy-independent GLUT transport, SGLTs, as active transporters, work against the glucose concentration gradient by coupling glucose transport to the downhill Na^+^ electrochemical gradient via Na^+^/K^+^-ATPase. Therefore, SGLT1 activation plays a particularly important role under pathological low-glucose conditions, such as that associated with ischemia, relative to stable conditions. The present finding of a larger K decrease in subjects with higher glucose levels during ischemic attacks suggests that SGLT1-Na^+^/K^+^ATPase coupling might be deeply involved in the pathophysiology of the acute phase of ACS. Given this possible link, in the present path models, we populated the data of the serum sodium levels as a possible variable for the determination of a decrease in K levels. Interestingly, when drawing the path model without including the sodium level, the standardized regression coefficient (β) was slightly reduced from 0.398 to 0.380 (the precise data was not shown). This result may also support our hypothesis that the sodium level provides to be important for glucose use during ACS.

The time of the last meal was not routinely documented for the patients presenting with ACS and may affect the data on glucose and insulin levels during ischemic attack in the present study. However, the sub-analysis with the subjects in “a definite fasting condition” clearly showed that the HOMA-IR was significantly increased, while the serum K level was decreased during ischemic attack. Although we only identified 23 patients as being in a definite fasting condition (confirmed by their medical history), there might have been more cases that were in a fasting state. However, we had to exclude such potential patients to ensure data accuracy, as we could not conclusively confirm that they were in “a definite fasting state”. Although the presence of various dietary conditions is a limitation associated with the present study, the HOMA-IR during both ischemic attack and the remission phase did not significantly influence the ΔK in any case, whereas the influence of the glucose level during attack on the ΔK was consistently significant (Fig 1 and Tables 3–5). In contrast, HOMA-β, which is in general dramatically elevated in the post-prandial state, actually turned out to be low-normal during ischemic attack (Table 2). Therefore, although the influence of the prandial state cannot be completely excluded in the current study, we cannot deny that insulin resistance was increased during ischemic attack.

A complex interaction of counter-regulatory hormones, such as catecholamines, cortisol and cytokines can cause the development of stress induced hyperglycemia [30]. The derangement of these neurohumoral factors antagonizes the action of insulin, promotes lipolysis, and increases circulating free fatty acid (FFA) levels, all of which can ultimately leads to high hepatic glucose output and insulin resistance [3, 30]. In this context, future studies are warranted to determine whether plasma glucose and insulin levels, as well as FFA level, are increased during ACS attack in comparison to both those before and after ischemic attack in order to more directly delineate “transient” insulin resistance, and to understand its potential mechanism. Moreover, it is possible that acute onset of other various serious diseases, such as heart failure, respiratory failure, or acute abdomen, may induce hyperglycemia by the above-described mechanisms. Thus, it would be interesting to see whether a transient increase in insulin resistance and a decrease in potassium level are observed in these clinical conditions. On the other hand, an increase in insulin resistance during ACS attack is of greater pathophysiological significance in comparison to an increase in insulin resistance during the acute phase of other serious diseases, given that promoting glucose metabolism, -which is an important preferential substrate for ischemic myocardium during acute phase of an ischemic attack- by GIK therapy has cardioprotective effects.

The sample size was somewhat small in the present study. However, covariance structure analysis as well as conventional multiple regression analysis consistently showed that glucose level was positively correlated with ΔK with the highest standard regression coefficient (Table 5 and Fig 1). Thus, considering the power of β, as shown in the covariance structure analysis (0.398 in path model [A] and 0.278 in [B]), we believe that ΔK is closely associated with glucose level during ACS attack, even under insulin-resistant conditions.

## Conclusions

Insulin resistance most likely increases during the acute phase of ACS attack. Although there is an endogenous GIK-tolerant state during ACS attack, a transient decrease in serum K level during ischemic attack was still observed, the degree of which was consistently positively correlated with glucose level but not correlated at all with HOMA-IR. These data suggest that, at the acute phase of ACS attack, there is “insulin-independent” intrinsic GIK cascade activation to promote glucose metabolism in order to cope with the critical ischemic condition, even in an insulin-resistant state. Meanwhile, the present study supports the findings of recent large clinical trials recommending that GIK administration be started as early as possible (namely, before a rapid increase in insulin resistance) [7, 11, 31, 32] and underscores the importance of preventing or ameliorating insulin resistance as an acute-phase treatment of ACS to ensure the effective utilization of the intrinsic GIK cascade activation.

## Supporting information

S1 Fig The time course of the plasma glucose and insulin level profiles. An individual patient’s profile (n = 104) is indicated by the arrow pointing to the data during remission phase from those during ischemic attack.
(TIFF)

S2 Fig The comparison of ΔK among the changes in the medication profiles. The comparison of ΔK among the indicated changes in the medication profiles of rennin-angiotensin-aldosterone system inhibitors (RAAS-I) (newly administered (-): n = 52; (+): n = 52) (**A**) and diuretics (newly administered (-): n = 96; (+): n = 8) (**B**) in all patients (n = 104) are shown. The definition of ‘newly administered’ is described in the *Methods* section. **(C)** The ΔK is compared among the medication profiles for β-blockers; (-) indicates the subjects who were not taking any β-blockers at admission (n = 86), and those with β1 selective β-blocker use (n = 10) and those with non-selective β-blocker use (n = 8) at admission are represented. **P<0.01 by independent *t*-test. NS; not significant.
(TIFF)

S3 Fig Receiver operating characteristic (ROC) curve for detecting ΔK threshold for the incidence of myocardial infarction. The cut-off value of ΔK is 0.3 with a combination of the highest sensitivity and specificity. AUC; area under the ROC curve.
(TIFF)

S4 Fig Path model theoretically proposed for the correlation between the effects of newly-administered angiotensin-aldosterone system inhibitors (RAAS-I) and the disease severity. Each path has a coefficient showing the standardized coefficient of a regressing independent variable on a dependent variable of the relevant path. These variables represent standardized regression coefficients (direct effect) [underlined portions indicate remarkable values] and squared multiple correlations [in narrow italics].
(TIFF)

S1 Table The comparison of the data during ischemic attack and remission phase in patients under fasting condition confirmed by medical history. (PDF)

S2 Table Medication profile. (PDF)

S3 Table The results of a multiple regression analysis of ΔK. (PDF)

S4 Table The results of a multiple regression analysis of ΔK. (PDF)

S5 Table The impact of ΔK on disease severity and clinical course based on quartiles of ΔK. (PDF)

S6 Table The results of standardized regression coefficient analysis to identify the correlation between the effects of RAAS-I newly administered and the disease severity in path model. (PDF)
