# Supplementary figures and images for: Possible increase in insulin resistance and concealed glucose-coupled potassium-lowering mechanisms during acute coronary syndrome documented by covariance structure analysis
Source: PLoS One. 2017 Apr 21;12(4):e0176435. doi: 10.1371/journal.pone.0176435 (PMC5400267; doi:10.1371/journal.pone.0176435)

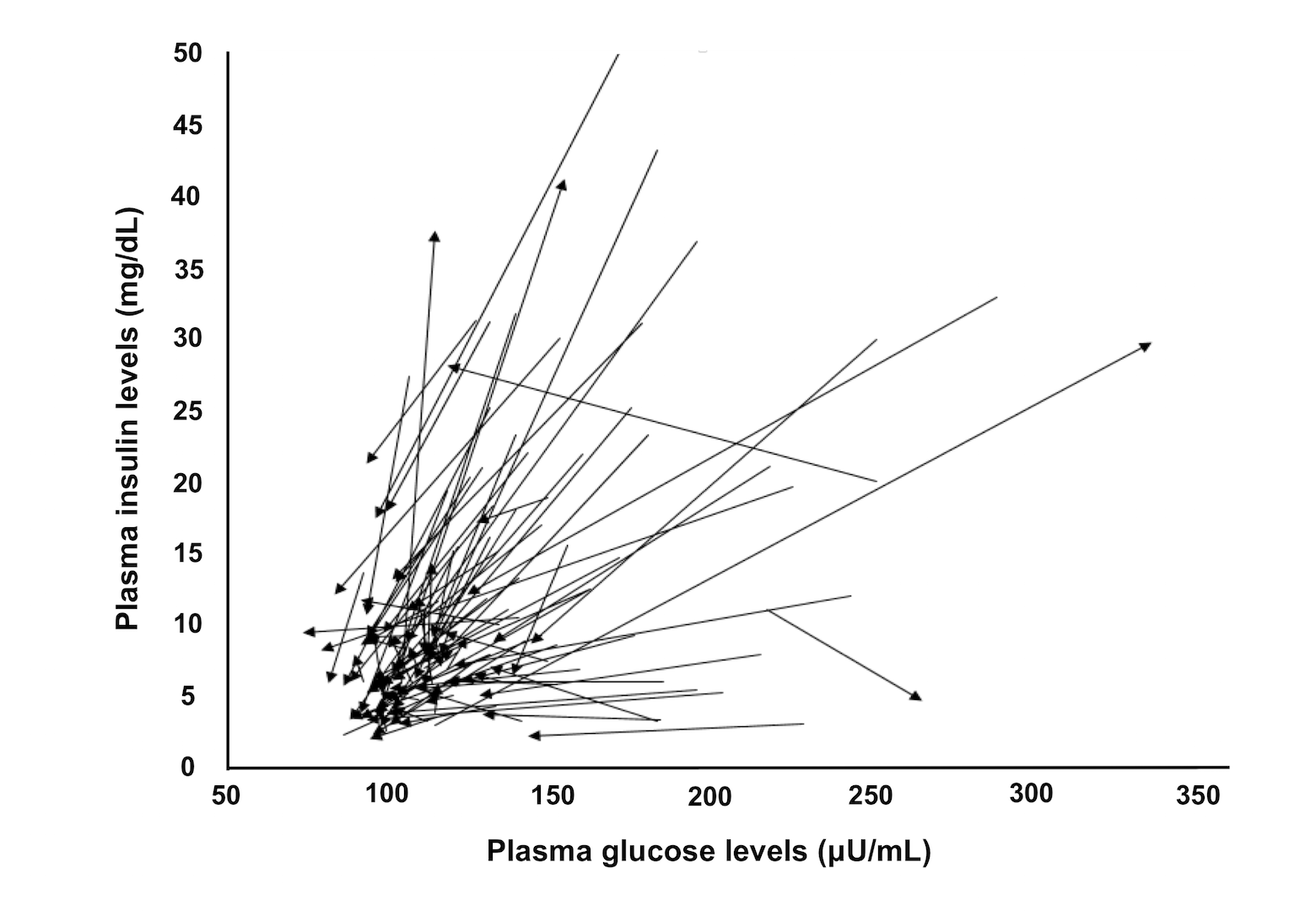

Supplement: S1 Fig — An individual patient’s profile (n = 104) is indicated by the arrow pointing to the data during remission phase from those during ischemic attack. (TIFF) [file pone.0176435.s001.tiff]

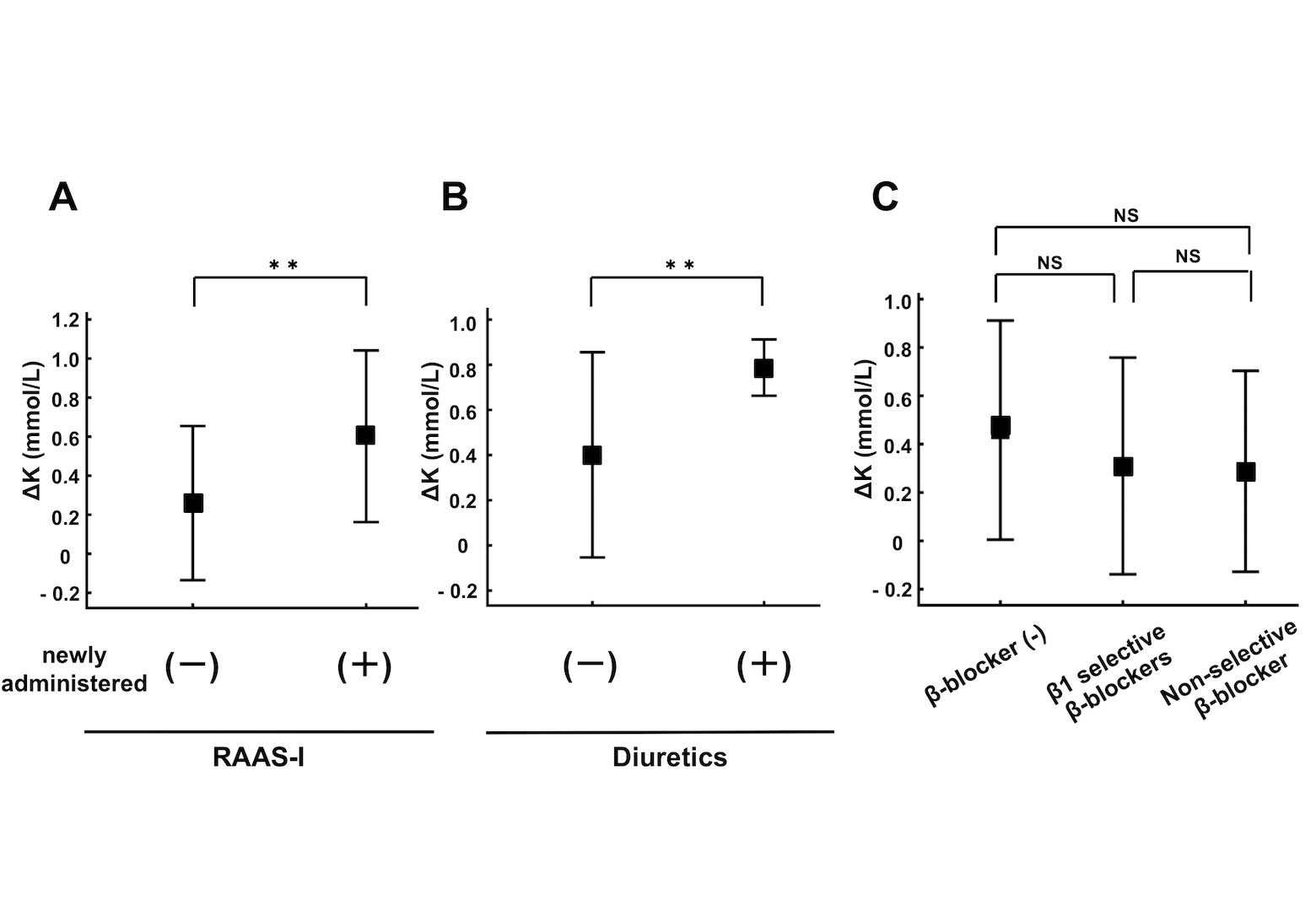

Supplement: S2 Fig — The comparison of ΔK among the indicated changes in the medication profiles of rennin-angiotensin-aldosterone system inhibitors (RAAS-I) (newly administered (-): n = 52; (+): n = 52) (A) and diuretics (newly administered (-): n = 96; (+): n = 8) (B) in all patients (n = 104) are shown. The definition of ‘newly administered’ is described in the Methods section. (C) The ΔK is compared among the medication profiles for β-blockers; (-) indicates the subjects who were not taking any β-blockers at admission (n = 86), and those with β1 selective β-blocker use (n = 10) and those with non-selective β-blocker use (n = 8) at admission are represented. **P<0.01 by independent t-test. NS; not significant. (TIFF) [file pone.0176435.s002.tiff]

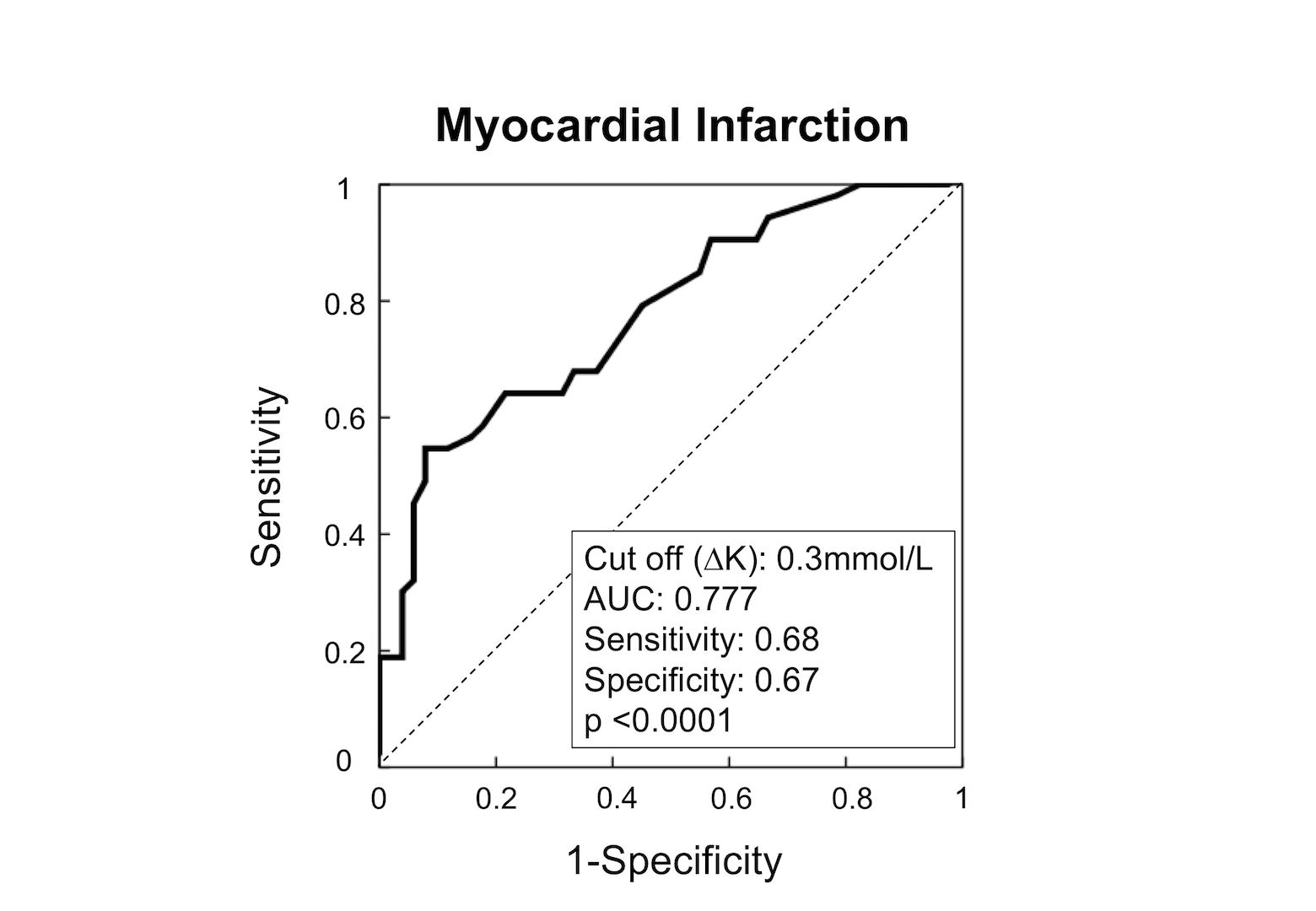

Supplement: S3 Fig — The cut-off value of ΔK is 0.3 with a combination of the highest sensitivity and specificity. AUC; area under the ROC curve. (TIFF) [file pone.0176435.s003.tiff]

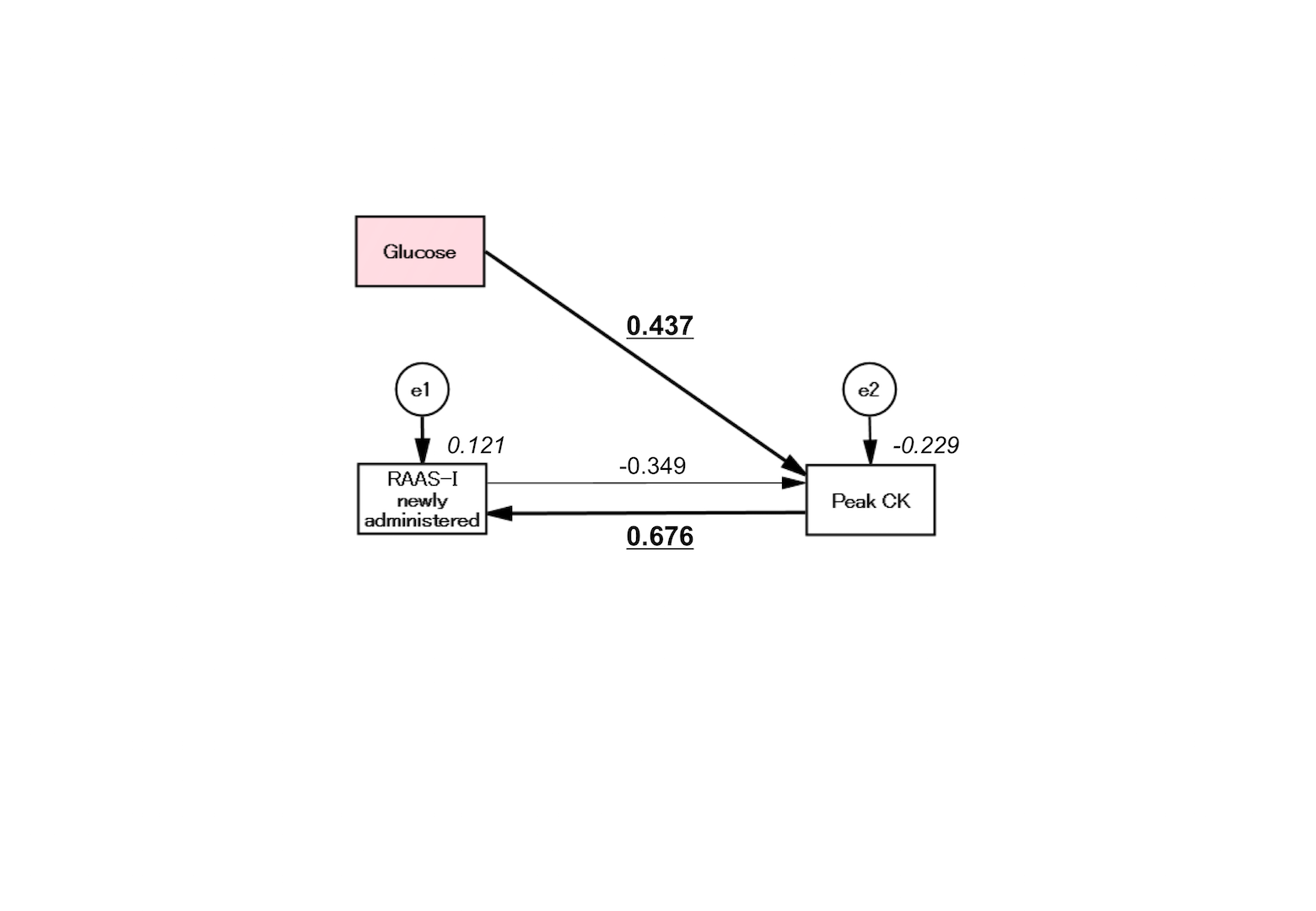

Supplement: S4 Fig — Each path has a coefficient showing the standardized coefficient of a regressing independent variable on a dependent variable of the relevant path. These variables represent standardized regression coefficients (direct effect) [underlined portions indicate remarkable values] and squared multiple correlations [in narrow italics]. (TIFF) [file pone.0176435.s004.tiff]
