# Supplementary material for: Possible increase in insulin resistance and concealed glucose-coupled potassium-lowering mechanisms during acute coronary syndrome documented by covariance structure analysis
Source: PLoS One. 2017 Apr 21;12(4):e0176435. doi: 10.1371/journal.pone.0176435 (PMC5400267; doi:10.1371/journal.pone.0176435)
Supplement: S1 Table — (PDF) [file pone.0176435.s005.pdf]

S1 Table. The comparison of the data during ischemic attack and remission phase in patients under fasting condition confirmed by medical history. (n=23)

	Ischemic attack	Remission phase	**P**
K, mmol/L	4.0±0.2	4.4±0.3	<0.001
Glucose, mg/dL	134.2±42.0	101.8±9.2	0.001
Insulin, μU/mL	13.6±8.1	7.7±3.3	0.001
HOMA-IR	5.1±5.1	2.0±1.0	0.004
HOMA-β	71.1±28.6	71.6±24.0	0.926
BNP, pg/mL	87.0±250.0	79.7±159.7	0.894

HOMA-IR: homeostasis model assessment of insulin resistance.

HOMA-β: homeostatic model assessment beta cell function.
