# Supplementary material for: Possible increase in insulin resistance and concealed glucose-coupled potassium-lowering mechanisms during acute coronary syndrome documented by covariance structure analysis
Source: PLoS One. 2017 Apr 21;12(4):e0176435. doi: 10.1371/journal.pone.0176435 (PMC5400267; doi:10.1371/journal.pone.0176435)
Supplement: S2 Table — (PDF) [file pone.0176435.s006.pdf]

S2 Table. Medication profile.

Drugs	Number on admission	Mean dose (mg/day)	Number at discharge	Mean dose (mg/day)
ACE-I	4		54	
Imidapril	1	2.5	9	3.3±1.3
Enalapril	1	5	41	3.2±1.3
Perindopril	1	2	3	2.7±1.2
Trandolapril	1	1	1	1
ARB	25		23	
Valsartan	8	75.0±14.1	2	80
Candesartan	3	8.0±4.0	0	
Losartan	3	50	2	37.5±17.7
Telmisartan	4	35.0±19.1	5	36.0±16.7
Olmesartan	4	25.0±10.0	7	20.0±10.0
Irbesartan	2	100	1	100
Azilsartan	1	20	6	23.3±8.2
Aliskiren	1	150	0	
Mineralocorticoid receptor inhibitors				
Spironolactone	2	25	17	23.5±4.2
Eplerenone	0		3	25
RAAS-I free	73		23	
β-blockers (non-selective)	18 (8)		60 (47)	
Carvedilol	8	6.9±3.5	47	3.7±2.2
Bisoprolol	7	2.5	13	2.0±0.6
Atenolol	2	25	0	
Celiprolol	1	100	0	

Loop diuretics	2		11	
Furosemide	2	30.0±14.1	10	23.0±9.5
Azosemide	0		1	15
Thiazide	5		2	
Hydrochlorothiazide	2	12.5	0	
Trichlormethiazide	3	1.7±0.6	2	1.5±0.7

ACE-I: angiotensin-converting-enzyme inhibitor, ARB: angiotensin-receptor-blocker, RAAS-I: renin-angiotensin-aldosterone system inhibitors
