# Supplementary material for: Possible increase in insulin resistance and concealed glucose-coupled potassium-lowering mechanisms during acute coronary syndrome documented by covariance structure analysis
Source: PLoS One. 2017 Apr 21;12(4):e0176435. doi: 10.1371/journal.pone.0176435 (PMC5400267; doi:10.1371/journal.pone.0176435)
Supplement: S3 Table — (PDF) [file pone.0176435.s007.pdf]

S3 Table. The results of a multiple regression analysis of ΔK (n=104).

Significant variables	Standard regression coefficients	Standard error	P
HOMA-IR during ischemic attack	0.077	0.009	0.439
HbA1c (NGSP)	-0.051	0.057	0.597
Na	0.062	0.018	0.524
eGFR	-0.005	0.002	0.959
LVEF	-0.230	0.005	0.038
RAAS-I newly administered	0.246	0.093	0.020
Diuretics newly administered	0.084	0.170	0.411

Dependent variable: ΔK

Explanatory variables: HOMA-IR, HbA1c, Na, eGFR, LVEF.

RAAS-I or diuretics newly administered.
