# Supplementary material for: Possible increase in insulin resistance and concealed glucose-coupled potassium-lowering mechanisms during acute coronary syndrome documented by covariance structure analysis
Source: PLoS One. 2017 Apr 21;12(4):e0176435. doi: 10.1371/journal.pone.0176435 (PMC5400267; doi:10.1371/journal.pone.0176435)
Supplement: S4 Table — (PDF) [file pone.0176435.s008.pdf]

S4 Table. The results of a multiple regression analysis of ΔK (n=104).

Significant variables	Standard regression coefficients	Standard error	P
HOMA-β during ischemic attack	-0.054	0.001	0.570
HbA1c (NGSP)	-0.044	0.057	0.642
Na	0.031	0.018	0.750
eGFR	0.003	0.002	0.978
LVEF	-0.238	0.005	0.031
RAAS-I newly administered	0.255	0.092	0.015
Diuretics newly administered	0.070	0.169	0.491

Dependent variable: ΔK

Explanatory variables: HOMA-β, HbA1c, Na, eGFR, LVEF.

RAAS-I or diuretics newly administered.
