# Supplementary material for: Possible increase in insulin resistance and concealed glucose-coupled potassium-lowering mechanisms during acute coronary syndrome documented by covariance structure analysis
Source: PLoS One. 2017 Apr 21;12(4):e0176435. doi: 10.1371/journal.pone.0176435 (PMC5400267; doi:10.1371/journal.pone.0176435)
Supplement: S5 Table — (PDF) [file pone.0176435.s009.pdf]

S5 Table. The impact of ΔK on disease severity and clinical course based on quartiles of ΔK

	ΔK < lower quartile	ΔK ≥ lower quartile	
Myocardial Infarction	3 (15.0%) [20]	50 (59.5%) [84]	P<0.001
Peak Creatine Kinase (U/L)	357.0±574.5 [20]	1452.1±1941.9 [84]	P=0.001
Duration of hospital stay (days)	6.1±3.7 [19]	10.9±6.8 [82]	P=0.001

Lower quartile value of ΔK = 0.1

	ΔK < upper quartile	ΔK ≥ upper quartile	
Myocardial Infarction	27 (36.5%) [74]	26 (86.7%) [30]	P<0.001
Peak Creatine Kinase (U/L)	729.8±1136.4 [74]	2503.6±2472.0 [30]	P<0.001
Duration of hospital stay (days)	9.2±7.1 [71]	11.8±4.6 [30]	P=0.002

Upper quartile value of ΔK = 0.7

The number of cases is noted in square brackets.
