# Supplementary material for: Possible increase in insulin resistance and concealed glucose-coupled potassium-lowering mechanisms during acute coronary syndrome documented by covariance structure analysis
Source: PLoS One. 2017 Apr 21;12(4):e0176435. doi: 10.1371/journal.pone.0176435 (PMC5400267; doi:10.1371/journal.pone.0176435)
Supplement: S6 Table — (PDF) [file pone.0176435.s010.pdf]

S6 Table. The results of standardized regression coefficient analysis to identify the correlation between the effects of RAAS-I newly administered and the disease severity in path model.

		Clinical factor	Direct effect	P
Peak Creatine Kinase	←	Glucose	0.437	0.002
	←	RAAS-I newly administered	-0.349	0.374
RAAS-I newly administered	←	Peak Creatine Kinase	0.676	0.010
